# Frequent Queen Replacement and Presence of Unrelated Queens in Colonies of a Functionally Monogynous Ant Species

**DOI:** 10.1002/ece3.71133

**Published:** 2025-05-26

**Authors:** Marion Cordonnier, Lena Bachl, Nicolas Thiercelin, Andreas Trindl, Jürgen Heinze, Abel Bernadou

**Affiliations:** ^1^ Chair of Zoology and Evolutionary Biology University of Regensburg Regensburg Germany; ^2^ Centre de Recherches sur la Cognition Animale, Centre de Biologie Intégrative Université de Toulouse, CNRS, UPS Toulouse France

**Keywords:** dominance hierarchy, genetic relatedness, haplotypes, *Leptothorax gredleri*, ovary development, social parasitism

## Abstract

In eusocial insects, social parasitism—the exploitation of the host's brood care behavior for survival and reproduction—can occur either within or between species. Parasitic queens invade host nests and aggressively replace the resident queen. While the adoption of conspecific queens is a common feature of species with multiqueen colonies (polygyny), the origin of parasitic founding is not fully understood. Functionally monogynous ants, in which nestmate queens establish social and reproductive hierarchies through biting and antennal boxing, may provide a link between peaceful adoption and social parasitism. In this study, we investigated whether alien queens can usurp colonies of the functionally monogynous ant *Leptothorax gredleri* . Ovary dissection of queens from 33 nests showed that multiple queens with developed ovaries can occasionally co‐occur in the same nest. Genetic analysis revealed frequent replacement of the dominant queens by relatives. Instead, alien queens rarely take over reproduction, suggesting a few occurrences of intraspecific social parasitism. However, the data suggest that alien queens without developed ovaries frequently invade nests without being eliminated. This suggests that alien queens are somehow prevented from reproducing and social parasitism is therefore limited in this species.

## Graphical abstract

In this study, we investigated whether alien queens can usurp colonies of the functionally monogynous ant *Leptothorax gredleri* . Using ovary dissection of queens and genetic analyses, we showed that multiple queens with developed ovaries can occasionally co‐occur in the same nest, with a frequent replacement of the dominant queens by relatives. Instead, alien queens rarely take over reproduction, but alien queens without developed ovaries frequently invade nests without being eliminated, suggesting that alien queens are somehow prevented from reproducing and social parasitism is limited in this species.

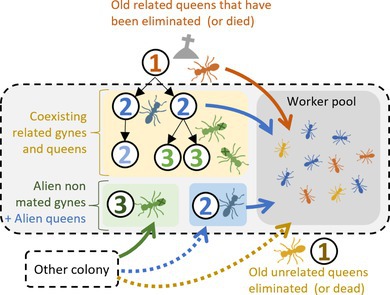

## 1 Introduction

In eusocial bees, ants, and wasps, numerous “social parasites” have evolved, which depend on the brood care behavior provided by workers from other colonies for survival and reproduction (Rabeling 2021). Social parasitism can occur both within (Caliari Oliveira et al. 2022) and between species (Cervo 2006; Buschinger 2009; Lhomme and Hines 2019). Ants (Formicidae) have for a long time been an important study subject for investigating social parasitism (e.g., Wheeler 1905; Emery 1909; Wasmann 1913). Recently mated queens of the parasite invade the nest of a related host species (or host colonies from the same species), where they either try to kill or expel the resident queen(s) or live alongside them. This usually has a detrimental effect on host fitness, as host workers are forced to take care of the parasite offspring.

The adoption of alien queens into conspecific polygynous colonies (i.e., with multiple reproductive queens) could be a reasonable start of queen‐tolerant inquilinism, where the parasitic queens produce no or few own workers but invest mainly in the production of sexuals (Sturtevant 1938; Buschinger 1965; Elmes 1973; Borowiec et al. 2021). In contrast, the origin of the often brutal behavior of workerless “murder‐parasites” (e.g., Faber 1969; Buschinger 2009), which kill the host queen by stinging, “throttling,” or cutting off its antennae or head, remains enigmatic.

Functionally monogynous ants, in which coexisting queens aggressively form social hierarchies, with only the most dominant individual laying eggs (Buschinger 1968; Heinze and Smith 1990; Heinze 1993), might provide a link between peaceful queen adoption and parasitic queen elimination in “murder‐parasites.” Functional monogyny and queen antagonism have been documented at least in some populations of several species of *Leptothorax* (Buschinger 1968; Heinze and Smith 1990; Buschinger and Francoeur 1991; Felke and Buschinger 1999; Ito 2005; Gill et al. 2009; Trettin et al. 2011; Heinze and Gratiashvili 2015), a myrmicine genus. It is well known from *Leptothorax gredleri* Mayr 1855 (Hymenoptera, Formicidae), a small Central European ant, which mostly nests in rotten twigs and under bark at the sunny edges of light pine‐oak forests, in rose or blackthorn thickets. Such habitat patches are quickly saturated, causing strong competition for nest sites and high reproductive skew (Bourke and Heinze 1994). Female sexuals (“gynes”) of *L. gredleri* leave their maternal nests in summer and release pheromones to attract males (“female calling”, Buschinger 1968; Heinze et al. 1992; Oberstadt and Heinze 2003). They shed their wings soon after mating and found a new nest solitarily, return to their natal nest, or potentially infiltrate an alien colony (Heinze et al. 1992; Bernadou et al. 2015). In the latter cases, multiple inseminated queens can be present in a colony (Buschinger 1968). After adoption and in particular after hibernation, nestmate queens establish dominance hierarchies by aggressive fighting, and only the top‐ranking queen begins to lay eggs (Buschinger 1968; Heinze et al. 1992). This differs strikingly from queen tolerance in related polygynous species. For example, in *Leptothorax acervorum* , egg eating rather than overt aggression seems to play a role in controlling reproduction (Bourke 1991, 1994).

Whereas in polygynous species, colonies may occasionally adopt alien queens (Evans 1996; Stille and Stille 1992; Zinck et al. 2009), in functionally monogynous species, this might lead to aggressive queen replacement and thus intraspecific parasitism (Buschinger 1990; Heinze and Lipski 1990; Bourke and Franks 1991). The aim of the present study was therefore to determine the genetic structure of multiqueen colonies of *L. gredleri* and to reveal whether alien queens may occasionally be adopted and successfully compete with resident queens for egg laying. We genotyped at least eight workers and all queens from 33 multiqueen colonies at eight microsatellite loci. Whether alien queens took over reproduction was assessed from colony structure, genetic relatedness among nestmate queens, and determination of the queens' haplotype of the mitochondrial DNA gene COI–COII. In addition, reproductive status was investigated by ovary dissection.

## 2 Materials and Methods

### 2.1 Sampling and Dissections

Fifty colonies of *L. gredleri* were collected near Erlangen, southern Germany (49.584167°, 11.030278°) between March 8th and April 11th, from 2011 to 2013. All queens and workers were counted and stored at −20°C. Among them, 17 colonies were either queenless (*n* = 4) or monogynous (*n* = 13) and have not been further investigated. The remaining 33 colonies contained between 2 and 15 queens (median 4) and were the subject of the present study.

Ovaries were dissected from each queen and photographed. In *L. gredleri* , ovaries typically have six ovarioles, with the spermatheca at their bases (Heinze et al. 1992). We categorized the queens as either egg layer when ovarioles were fully elongated (mean ovarioles length = 3.433 ± 1.059) and yellow bodies (remnants of previously laid eggs) were present, or nonlayer, when ovarioles were not elongated (mean ovarioles length = 1.083 ± 0.201) and contained only a few developing eggs (modified from Heinze et al. 1992). The presence of sperm in the spermatheca was not used as a relevant indicator to determine the queen's status, as the binary decision (inseminated vs. not inseminated) was difficult based on the pictures and could have resulted in a potential bias in the results (Figure S1)—and because even with an empty spermatheca, ovaries can be developed, and the queen can produce males. While the number of fertile eggs is sometimes used as a fertility indicator (e.g., Kühbandner et al. 2014), this criterion was not relevant here as queens were collected at the beginning of the reproduction period when few eggs were present.

Detailed information on colony composition and reproductive status of queens is available on the Zenodo repository (https://doi.org/10.5281/zenodo.14826017).

### 2.2 DNA Extraction and Microsatellites Data

DNA was extracted from the head and mesosoma of all queens and from the whole body of eight workers per colony (*n* = 33) using a CTAB method (cetyltrimethylammonium bromide; modified from Sambrook and Russell 2001). This resulted in a total of 426 genotyped individuals (273 workers and 153 queens; note that two colonies contained less than 8 workers, and in two colonies, all their 17 and 19 workers were genotyped to verify the accuracy of the estimates; Table S1 for details).

Following DNA extraction, all individuals were genotyped at eight highly variable microsatellite loci: LXAGa1, LXAGa2 (Bourke et al. 1997), L‐18 (Foitzik et al. 1997), LXGT218, LXGT223 (Hamaguchi et al. 1993), 2MS34 (Suefuji et al. 2011), 2MS46 (M Suefuji, unpublished), and Ant10878 (Butler et al. 2014) (primer sequences available in Table S2).

The 10 μL PCR reaction volume consisted of 5 μL buffer with Taq DNA polymerase (Promega M7433 Taq G2 Hot Start ColorlessMaster Mix), 2 μL ddH_2_O, 1 μL unlabeled reverse primer (10 μM), 1 μL labeled forward primer (10 μM; HEX, FAM, and TET), and 1 μL DNA (2–10 ng). The PCR involved an initial denaturation at 94°C (3 min), 33 cycles at 94°C (denaturation, 30 s for 2MS34 and 2MS46, 45 s for the other primers), 55°C (2MS34, 2MS46) or 54°C (all other primers) (annealing, 30 s) and 72°C (elongation, 30 s), and a final step at 72°C (1 min). The PCR products were analyzed on an ABI PRISM 310 Genetic Analyzer (PE Biosystems) after DNA denaturation at 90°C (1 min). Allele sizes were determined using GeneScan 3.1 software (PE Biosystems). The molecular analyses were repeated until at least seven successful loci were obtained for all individuals. All eight loci were polymorphic and showed substantial variation, with an average of 11.6 alleles across all samples (LXAGa1: 5 alleles, LXAGa2: 22, L‐18: 12, LXGT218: 4, LXGT223: 23, 2MS34: 14, 2MS46: 8, Ant10878: 5). To assess marker quality, the genotype from one worker per colony was used to check the heterozygote deficiency and linkage disequilibrium using the software GenePop (Raymond and Rousset 1995; Rousset 2008). The absence of scoring errors due to stuttering, large allele dropout, or null alleles was checked using the software Microchecker (Van Oosterhout et al. 2004; Tables S3 and S4). The complete genotypes are available on the Zenodo repository (https://doi.org/10.5281/zenodo.14826017).

In one of the colonies, triploidy has been detected in all eight workers and one of the queens, suggesting the rare (and until now unreported) existence of diploid males in this species, as documented for other ant species ( *Solenopsis invicta* , Krieger et al. 1999; *Tapinoma erraticum* , Cournault and Aron 2009). This colony was investigated like the other ones, but we used only the smaller of the two alleles for relatedness calculation (leading to discard only four alleles in total).

### 2.3 Mitochondrial Sequencing

All the 153 queens were Sanger sequenced for a stretch of mitochondrial gene covering the genes cytochrome oxidase I and II (CO I and II) including the tLeu region (Simon et al. 1994; Table S2). The 25 μL PCR reaction volume consisted of 12.5 μL buffer with Taq DNA polymerase, 9.5 μL ddH_2_O, 1 μL reverse primer, 1 μL forward primer (final concentration of 0.5 μM), and 1 μL DNA (2–10 ng). The PCR consisted of an initial denaturation at 94°C (4 min), 37 cycles at 94°C (denaturation, 45 s), 50°C (annealing, 45 s) and 72°C (elongation, 60 s), and a final step at 72°C (5 min). PCR products were cleaned up using the NucleoSpin Gel and PCR Macherey‐Nagel's Clean‐up Kit, and sequencing was conducted by LCG Genomics GmbH (Berlin, Germany). Forward and reverse DNA sequences of the same individual were proofread against each other and corrected with Chromas 2.23 and BioEdit 7.0.5 (Hall 1999). Sequences from four queens were not considered to have sufficient quality and were excluded from the analysis, corresponding to 149 sequences incorporated in the study (GenBank accession numbers [LN831904–LN831907]).

The definition of four mitochondrial haplotypes (A, B, C, D) was based on consensus sequences of the COI and COII sequences, corresponding to fragments of 820 bp length. The sequences were aligned using ClustalW (Thompson et al. 1994) as implemented in BioEdit. Statistical parsimony haplotype network was constructed with TCS 1.21 (Clement et al. 2000).

### 2.4 Genetic Structure of the Colonies

Worker genotypes were used to reconstruct the genotypes of their mothers, that is, at each locus, the two alleles shared among all workers were assigned to the queen. Since the species is expected to be monandrous (i.e., queens are singly mated; Oberstadt and Heinze 2003), the remaining unassigned alleles were attributed to the haploid fathers. This process was repeated for all eight microsatellite markers while minimizing the number of queens per colony (Cordonnier et al. 2020, 2022). Genotypes were reconstructed based on a minimum of three offspring individuals, which could be either workers or other queens. If fewer than three offspring were associated with a queen, genotype reconstruction was not considered sufficiently reliable, and only the minimum number of additional queens required to explain the genotypes was recorded. The inferred genotypes of the queens were compared to the actual genotypes of the collected queens. This aimed to identify matches between collected queens and the reproductive queen(s) contributing to the offspring within each colony. Additionally, the genotypes of the collected queens were tested to determine if they were relatives of the identified reproductive queens. Note that only collected queens with developed ovaries were identified as having offspring, confirming the reliability of the method (Table S1).

This method was preferred to the use of automated software because it required no assumptions about the mating system and no specification about which individuals were putative breeders and which were not. It gives the possibility to separate a posteriori the queens with and without developed ovaries, making this approach more suitable for a complex system as observed in *L. gredleri* . Although time‐consuming, such an approach also minimizes errors compared with classically used software (e.g., Colony, Matesoft) where data monitoring and analysis are automated, often without postprocess quality control. Here, scoring errors were checked and corrected one last time after maternal and paternal identification to avoid overestimating the number of potential breeders, making it less likely that the detection of breeders was caused by errors during sequencing or the determination of allele sizes.

The number of eight worker genotypes for the reconstruction of parental genotypes has been chosen based on the fact that this allows the detection of all breeders (among three active breeders) in about 90% of the cases, even in situations of unequal contribution to the offspring. In fact, in colonies with two egg‐laying queens and an equal contribution to the offspring, eight worker genotypes are sufficient to detect both queens in more than 99% of the cases. In colonies with two queens but an unbalanced offspring ratio (75/25%), both queens will be detected in more than 89% of the cases. In colonies with three reproductive queens and equally shared contribution to the offspring, they will be detected in more than 88% of the cases (see Table S5 for details and calculations). Note that in case a very unbalanced offspring ratio, for example, 90/10%, or when more than three queens contribute to the offspring, a small increase in the number of worker genotypes will not significantly improve the detection of the breeders.

To facilitate comparison between nests, the nest genetic structure has been encoded to distinguish among the following categories: collected queens with (un)developed ovaries, past breeders (former mothers based on the genotypes of offspring), and “extra” queens where genotype reconstruction was not possible. This coding system also accounts for the associated offspring among workers and queens, including the ovaries development of the latter (i.e., queens with developed and undeveloped ovaries). Colony composition was reconstructed independently by two persons (L.B. and M.C.) and subsequently compared to determine the most likely nest structure (see hereafter for details). For 31 out of the 33 colonies, both persons arrived at identical scenarios, which emphasizes the quality of the methods and the reliability of the results. However, for two colonies (ID 63 and 70), the high degree of relatedness among queens and the large number of collected queens resulted in many possible scenarios. Consequently, these two colonies were excluded from further analyses to prevent any overinterpretation of the results.

Based on the colony composition found (see Table S1 for details), three types of queens (1–3) and nests (A–C) were defined (Table 1).

**TABLE 1:** Definition of the three types of queens identified in the colonies, and the three types of nests depending to the genetic structure evidenced.

**Queen type**	
1	“Former mothers,” which have not been collected but whose genotype were built based on the genotypes of their offspring
2a	Collected queens with developed ovaries with offspring present in the nest
2b	Collected queens with developed ovaries without offspring present in the nest
3	Collected queens with undeveloped ovaries
**Nest type**	
A	Only one queen contributed to the offspring (queens from type 1 or 2; functional monogyny)
B	Several queens present in the nest contributed to the offspring (queens from type 1 and/or 2; polygyny)
C	Several queens contributed to the offspring, but maximum one of them was present in the nest (1 or more queens from type 1, and max 1 queen from type 2)

### 2.5 Statistical Analyses

Pairwise relatedness among workers and queens was calculated using the Queller and Goodnight estimator (1989) as implemented in Genalex (Peakall and Smouse 2006). The relative relatedness between pairs of queens within nests (*n* = 449 pairs, available on the Zenodo repository [https://doi.org/10.5281/zenodo.14826017]) was compared among the three types of queens, leading to six categories of queen pairs (type 1–type 1, type 1–type 2, …, type 3–type 3) (but note the same analysis was also conducted by separating type 2a and type 2b—Figure S2).

The relatedness estimates of nestmates within the six different categories of queens were analyzed for unimodality/multimodality using Hartigans' dip test. Because of the non‐normality of several of the categories, nonparametric pairwise Wilcoxon rank sum tests (with Holm correction) were conducted to assess differences between all pairs of categories (*n* = 15 tests).

Three (generalized) linear models were used to investigate if the nest type (independent variable) impacted (i) the number of collected queens with developed ovaries (model 1, generalized linear model, Poisson family), (ii) the total number of workers in the nest (model 2, linear model), and (iii) the average relatedness of nestmate workers (model 3, linear model). Pairwise post hoc comparisons (including a Holm correction) between all pairs of categories were performed using *t*‐tests and based on a self‐designed contrast matrix (package *emmeans*, Lenth et al. 2019). The validity of the models was checked based on the residual diagnostics (package *DHARMa*, Hartig 2022). For all statistical tests, the level of significance was set at *p* < 0.05. All statistical analyses were performed in R (R Core Team 2022).

## 3 Results

**FIGURE 1:**
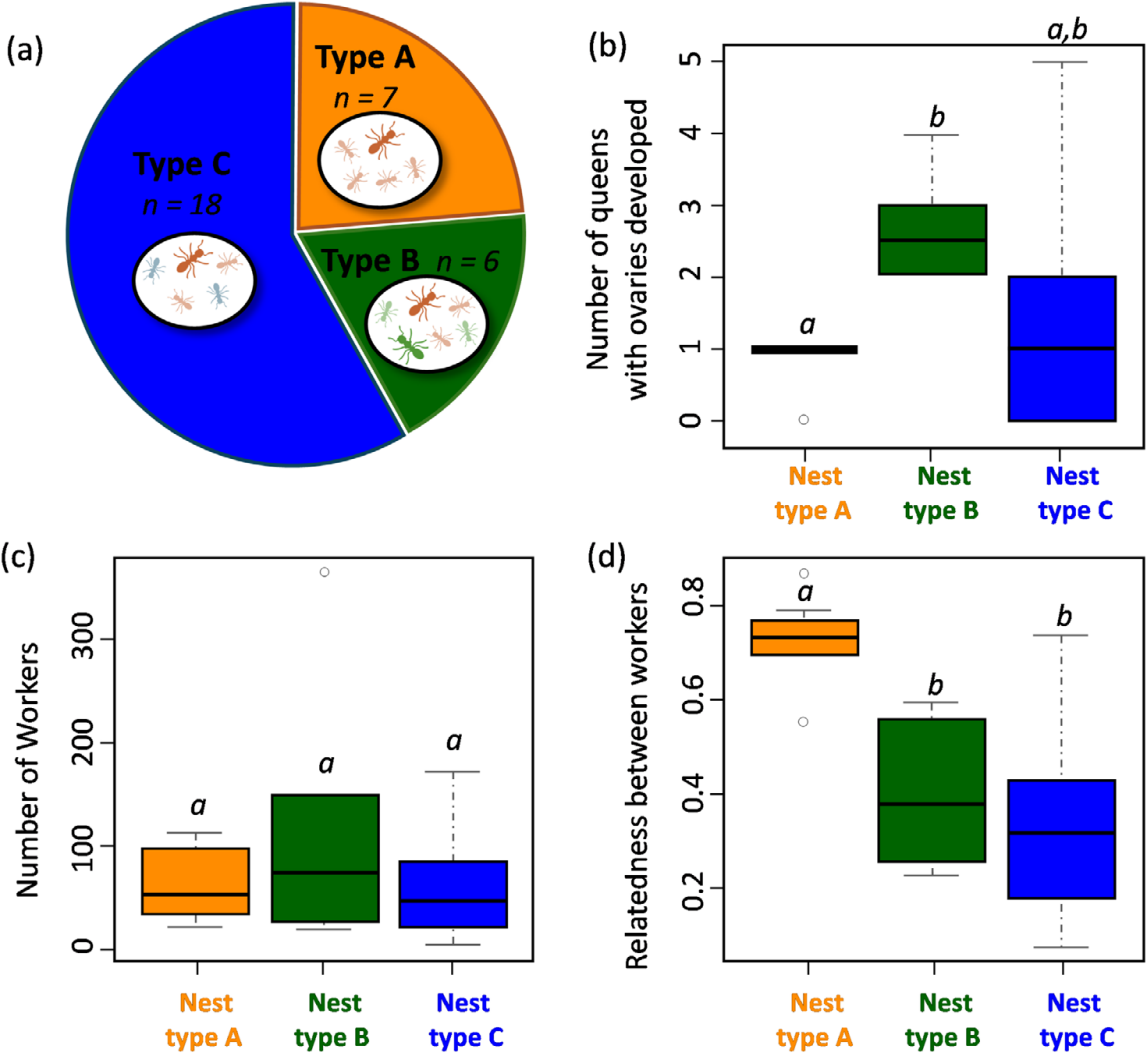
(a) Nest types identified in the study. Nest type A (orange): Only one queen contributed to the worker offspring; nest type B (green): Several queens contributed to the workers offspring and were present in the nest; nest type C (blue): Several queens contributed to the offspring but maximum one active queen was present in the nest. (b) Number of queens with developed ovaries in the three nest types. (c) Number of workers per nest in the three nest types. (d) Relatedness among nestmate workers in the three nest types. Significant differences are indicated with different letters (see Table S6 for details).

Previous studies in the ant *L. gredleri* have shown that a single dominant queen monopolizes reproduction even when multiple queens are present in a colony (Buschinger 1968; Heinze et al. 1992; Oberstadt and Heinze 2003). In contrast, our dissections revealed the presence of a single dominant egg layer in only 12 of the 33 multiqueen colonies studied. Instead, in 14 colonies, up to 11 queens had elongated ovaries (median 3), and seven colonies lacked an active egg layer at the time of sampling (Figure 1, Table S1).

While apparent polygyny may be a transient stage during hierarchy establishment (e.g., Heinze et al. 1992), genotyping of eight nestmate workers revealed that the structure of colonies was often much more complex than expected from functional monogyny: workers were offspring of one to nine (median 3) mother queens per nest (Table S1). Genotyping the entire set of workers in two colonies confirmed that eight workers were sufficient to reliably determine the main contributing queens. No evidence of polyandry was found, but the results highlighted triploidy in eight workers and one queen with undeveloped ovaries (sister of the workers) in one colony, suggesting the fertility of diploid males.

**FIGURE 2:**
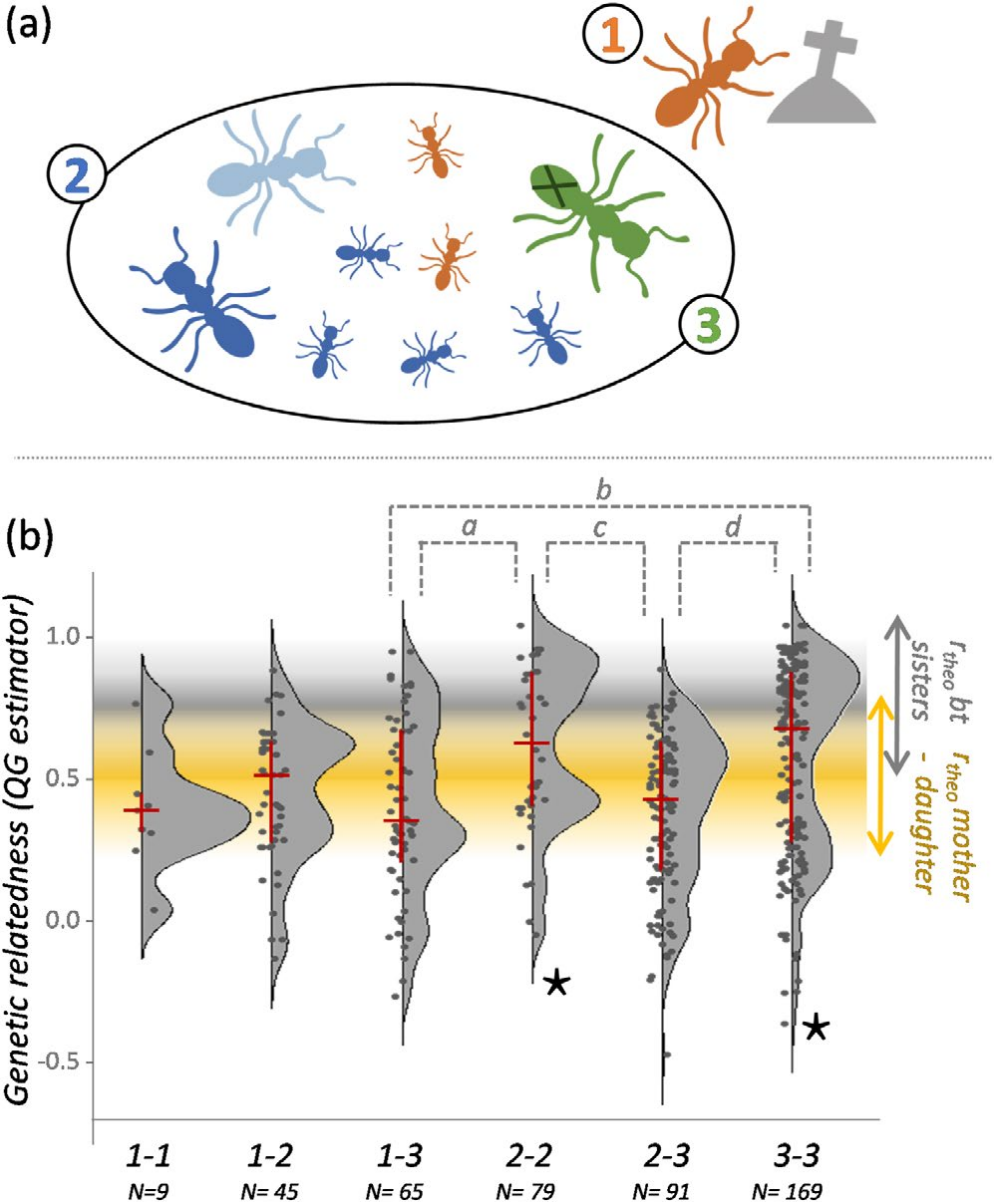
(a) Queen types identified in the study: (1) former mothers, not present in the nest; (2) collected queens with developed ovaries; (3) collected queens with undeveloped ovaries (indicated by a cross). (b) Genetic relatedness calculated between pairs of queens from the same nest. Red lines indicate quantiles (0.25–0.75) and median (horizontal line). Significant differences between type of comparisons are indicated in gray. a: *W* = 732, *p* = 0.009; b: *W* = 3558, *p* = 0.009; c: *W* = 3500, *p* = 0.002; d: *W* = 6769.5, *p* < 0.001. The stars indicate distributions significantly deviating from unimodality. The ranges on the right correspond to the theoretical relatedness values for full sisters (peaking at 0.75) and mother–daughter pairs (peaking at 0.5).

In almost half of the colonies (16 of 33), only one of the collected queens was identified as the mother of the workers. However, in six colonies, two to four of the collected queens contributed to the workers. In the remaining 11 colonies, none of the collected queens was the mother of the workers. Based on the maternal lineages found within each colony, three nest types were defined (see above). Seven colonies belonged to nest type A (only one queen contributed to the offspring), six colonies to nest type B (at least two present queens contributed to the offspring), and 18 colonies to nest type C (multiple queens contributed to the offspring but at most one was present in the nest) (Figure 2a).

As expected, the number of queens with developed ovaries was always 1 in nest type A (except for one nest where no queen with developed ovaries was found), and was therefore significantly lower than the number of queens in nest type B (median = 2.5, Figure 2b, Table S6). The number of queens with developed ovaries in nest type C was more variable than in the other nests (Figure 2b), but not significantly different from the other two nest types (Table S6).

The different nest types did not differ significantly in worker number (Table S6, Figure 2c). However, worker relatedness within nest type A was significantly higher (median of 0.754 corresponding to the theoretical full‐sister relatedness) than in types B and C (Table S6, Figure 2d).

Pairwise relatedness estimates differed among queen types (Figure 2, Table S7). While relatedness within and between most queen types displayed a unimodal distribution (type 1–type 1: *D* = 0.090, *p* = 0.778; type 1–type 2: *D* = 0.060, *p* = 0.186; type 1–type 3: *D* = 0.037, *p* = 0.802; type 2–type 3: *D* = 0.026, *p* = 0.846), we found a bimodal distribution for both queens with developed ovaries and those with undeveloped ovaries (type 2–type 2: *D* = 0.087, *p* = 0.015; type 3–type 3: *D* = 0.046, *p* = 0.013; Figure 2).

Pairwise relatedness suggested that nestmate queens with developed ovaries were mostly sisters and mothers/daughters. Only in two colonies (34 and 71) did unrelated queens (*r* < 0.25) with developed ovaries coexist, and only in colony 71 several of these unrelated queens had produced offspring. In contrast, queens without developed ovaries were often sisters and unrelated individuals (Figure 2b, Table S7). The presence of unrelated individuals in the group of type 3 queens (collected queens with undeveloped ovaries) is confirmed by the very low relatedness observed between some of the queens with developed and undeveloped ovaries (Figure 2b, type 2–type 3 comparison). Former mothers (type 1) were mostly related to the queens present in the colony, with a median relatedness indicating a mother–daughter filiation (Figure 2b, type 1–type 2 comparison). However, in a few cases, former mothers and collected queens appeared to be unrelated (e.g., 11 pairs of collected queens and former mothers had a relatedness < 0.3, of which three had a relatedness < 0.2).

Mitochondrial analysis of all collected queens revealed four different haplotypes (Figure S3). The number of queens per haplotype varied considerably, ranging from 3 (haplotype A) to 72 individuals (haplotype B) (haplotype C: 65 queens, haplotype D: 3 queens). Two colonies (colony 39 and colony 61, Table S1) contained queens from multiple lineages. In both cases, the “alien” queen (i.e., the queen with the less frequent haplotype) had undeveloped ovaries, confirming the genotype‐based findings that unrelated queens with developed ovaries were rarely found in the same nest. However, both the native and the alien queens were inseminated in colony 39 (but note this nest was constituted by only two queens and two workers in total).

## 4 Discussion

Our analysis of the colony structure of the functionally monogynous ant *L. gredleri* yielded two important results. First, the genetic composition of many colonies contained workers that were not offspring of a single queen but of queens that were no longer present and, occasionally, also of multiple present queens. This indicates frequent queen replacement and at least temporary polygyny. Second, the occurrence of queens that were unrelated to other nestmates makes intraspecific parasitism a likely option for colony founding.

In almost 20% of the colonies sampled for the current project, several queens with developed ovaries coexisted and produced worker offspring. This result differs from previous studies (Buschinger 1968; Heinze et al. 1992; Oberstadt and Heinze 2003), in which queen dissection had revealed that only a single queen reproduced per colony. As the nests studied here were collected in early spring, at a time when some nests may have been in the process of hierarchy formation among queens, it is difficult to know whether the coexistence of queens is transient or stable. The observation of workers being offspring of different queens suggests that the presence of multiple egg‐layers is not the result of recent queen readoption. Alternative explanations could be either colony fusion, that is, two colonies merging for hibernation, or pleometrosis, that is, temporary polygyny due to joint colony founding by queens. Both these phenomena are common in ants but have not yet been documented in *L. gredleri* . In related monogynous species, such as *Temnothorax nylanderi* and *Temnothorax crassispinus*, colony fusion between two unrelated colonies is common in areas with nest site limitation and leads to temporary polygyny. However, in such cases the second queen is typically eliminated within a few days after fusion, and only one queen resumes reproduction (Pusch et al. 2006). Similarly, in pleometrotic founding associations, reproduction is usually monopolized by one queen after the first workers have emerged (Bartz and Hölldobler 1982; Tschinkel and Howard 1983). Both explanations are therefore unlikely in the case of co‐occurring lineages in *L. gredleri* colonies.

Instead, functional monogyny resulting from social dominance hierarchies may not be as complete as previously thought. Indeed, colony structure varies among populations of the closely related *L. acervorum* , with colonies in areas with limited, patchily distributed nest sites being functionally monogynous and colonies in areas with more or less homogeneously distributed nest sites being facultatively polygynous (Ito 1990; Heinze and Ortius 1991; Bourke and Heinze 1994; Felke and Buschinger 1999; Gill and Hammond 2011). Further studies on the distribution and density of colonies with high and low reproductive skew in our study site may help to better understand the unexpected occurrence of multiple breeders in colonies. In addition, polygynous colonies in this study were slightly larger than functionally monogynous ones, perhaps indicating that it is less easy for a single queen to control reproduction in larger nests. Alternatively, colonies may simply become larger when multiple queens lay eggs.

**FIGURE 3:**
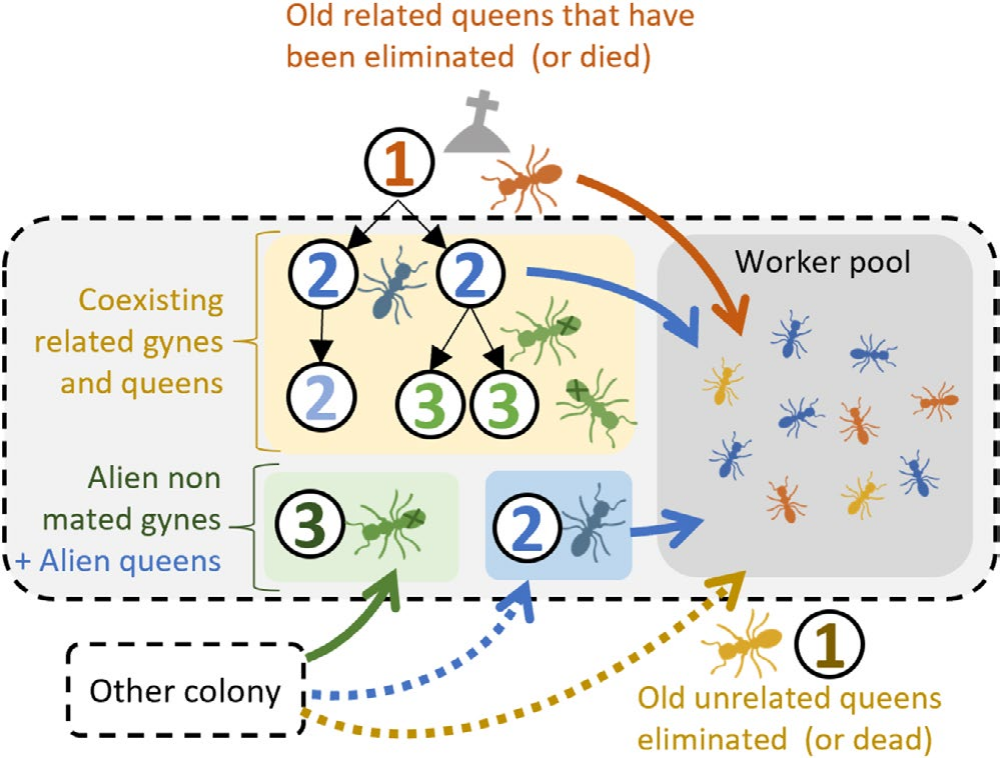
Summary of the main mechanisms likely to generate the observed patterns of colonies structure. The black dotted box indicates the outlines of the colony. (1) Former mothers, not present in the nest; (2) queens with developed ovaries; (3) queens without developed ovaries. Arrows indicate a direct parentage link. The colors in the worker pool correspond to the associated pool of queens.

In addition, our study documented that many colonies contained workers with genotypes of queens that were no longer present at the time of collection, suggesting queen replacement. This is consistent with previous observations that young queens can occasionally take over colonies (Heinze and Smith 1990; Heinze et al. 1992). According to the relatedness estimates, most of the queens were related. The results therefore converge toward a prevalent strategy (summarized in Figure 3) consisting of the turnover of related queens over time, that is, the new queens are daughters of the old queens. This result is consistent with the observations of Oberstadt and Heinze (2003), which suggest that mating takes place in the vicinity of the nests from which female sexuals originate. The considerable relatedness of nestmate queens was also highlighted by Heinze (1995) and confirms that they can return to their maternal nests after mating nearby.

Nevertheless, the genetic data also revealed the occurrence of queens that were unrelated to their nestmates. Although unrelated queens with developed ovaries (either with or without offspring) were rare, two colonies contained queens with different haplotypes. Unrelated queens may have been under‐detected because of the limited number of workers genotyped and the difficulty of reconstructing queen genotypes when too few offspring had been sampled. This may have biased the relatedness pattern and prevented the detection of the past replacement of queens by unrelated individuals, thus underestimating the frequency of social parasitism. However, the frequent presence of unrelated queens with undeveloped ovaries (i.e., nonmated queens or nonlaying queens—green arrow in the Figure 3) suggests that colonies often accept unrelated queens. While we did not find co‐occurring unrelated, egg‐laying queens, several nests contained unrelated nonlaying queens or related laying queens. This may indicate that colonies accept nonrelated queens as long as they do not compete for egg laying. The fact that alien queens of *L. gredleri* frequently enter nests of unrelated colonies, where they only occasionally reproduce without replacing the old queen, is of major interest. Acceptance of alien nonlaying queens is uncommon in monogynous species (Meunier et al. 2011; Sundström 1997), and the difference between the numbers of inseminated and uninseminated alien queens indicates that both origin and fertility status of gynes and queens play a role in the adoption process. The fitness benefits for adopted alien queens with undeveloped ovaries are not clear, as queen replacement seems to occur mainly among relatives, so the chance to lay eggs is very low for these individuals. Further studies investigating whether these alien gynes produce males or mate in the adopting colony would be of major interest. Adopting alien gynes would be beneficial to both source and recipient colonies if the sexuals mated in close proximity to or even inside the nest (Vidal et al. 2021). This raises the question of the occurrence of intranidal mating in *L. gredleri* and highlights the importance of further research at the intersection between social evolution and reproductive biology of this species.

## Author Contributions

**Marion Cordonnier:** data curation (equal), formal analysis (lead), methodology (equal), supervision (equal), writing – original draft (equal), writing – review and editing (equal). **Lena Bachl:** data curation (equal), formal analysis (equal), methodology (equal), writing – original draft (equal). **Nicolas Thiercelin:** data curation (supporting), formal analysis (supporting), methodology (supporting), writing – review and editing (supporting). **Andreas Trindl:** data curation (equal), methodology (equal), writing – review and editing (supporting). **Jürgen Heinze:** conceptualization (equal), funding acquisition (equal), supervision (equal), writing – original draft (equal), writing – review and editing (equal). **Abel Bernadou:** conceptualization (equal), funding acquisition (equal), methodology (equal), supervision (equal), writing – original draft (equal), writing – review and editing (equal).

## Disclosure

Benefit‐Sharing Statement: Benefits from this research accrue from the sharing of our data and results on public databases as described above.

## Ethics Statement

No ethical oversight is required to work with these animals. However, note that we collected the minimum of biological material needed for the study. The sampling was conducted cautiously to prevent any impact at the population level.

## Conflicts of Interest

The authors declare no conflicts of interest.

## Supporting information


Appendix S1

## Data Availability

The genotype raw data generated during the current study, the information about colonies and ovaries dissection, and the relative relatedness between pairs of queens within nests are available in the Zenodo repository (https://doi.org/10.5281/zenodo.14826017). The sequences are available in GenBank (accession numbers LN831904–LN831907).
