# Supplementary material for: Frequent Queen Replacement and Presence of Unrelated Queens in Colonies of a Functionally Monogynous Ant Species
Source: Ecol Evol. 2025 May 26;15(5):e71133. doi: 10.1002/ece3.71133 (PMC12106350; doi:10.1002/ece3.71133)
Supplement: Supplementary file 1 — Appendix S1 [file ECE3-15-e71133-s001.docx]

**Supplemental Information for:**

**Colony structure and apparent intraspecific social parasitism**

**in the functionally monogynous ant *Leptothorax gredleri***

Marion Cordonnier^1^*, Lena Bachl^1^, Nicolas Thiercelin^1^, Andreas Trindl^1^, Jürgen Heinze^1^†, Abel Bernadou^2^†

1. Chair of Zoology and Evolutionary Biology, University of Regensburg, Regensburg, Germany
2. Centre de Recherches sur la Cognition Animale, Centre de Biologie Intégrative, Université de Toulouse, CNRS, UPS, France

*Corresponding author, email: marion.cordonnier@hotmail.fr

†Jürgen Heinze and Abel Bernadou contributed equally to this work.

**Table of Contents:**

| **Table S1. Complete structure of the colonies studied** | Page 2 |
| --- | --- |
| **Table S2. Primer sequences**  **Table S3. Quality analysis of the microsatellite markers** | Page 3 |
| **Table S4. Linkage disequilibrium between microsatellite markers**  **Table S5. Detection probabilities of breeders** | Page 4 |
| **Table S6. Results of the models**  **Table S7. Pairwise Wilcoxon rank sum tests**  **Figure S1. Content of the queens spermatheca** | Page 5 |
| **Figure S2. Different types of queens identified in the study and**  **genetic relatedness between them**  **Figure S3. Maximum parsimony spanning network** | Page 6 |

**Table S1.** Complete structure of the colonies studied. ColID: identity of the colony; NW: number of collected workers; NQ: number of collected queens; NDQ: number of queens with fully developed ovaries; NPDQ: number of queens with partly developed ovaries; NPM: number of putative mothers as estimated from worker genotypes (min), NQM: number of collected queens identified as mothers; NQWS: number of collected queens identified as workers' sisters. Code is noted Ac/b/p(y)[Bx(y)][Cx]+ Ac/b/p(y)[Bx(y)][Cx]+…+Dx . Queens were categorized as Ac (“collected” queen with developed ovaries (stage 3 of ovarian development), Ab (absent queens based on the genotypes of three or more offspring, i.e., past breeders), or Ap (“phantom” queen, where genotype reconstruction was not possible as only one or two offspring were found). Associated with each A were “B” and “C” coding for collected queens’ daughters of the queen A with developed (i.e., stage 3) and undeveloped (i.e., stages 0 to 2) ovaries, respectively (with x indicating the number of queens concerned). For each collected queen, former mother or phantom queen, the number of worker offspring was indicated in round brackets (y). Additional contributing queen were separated with a “+” sign. Collected queens with undeveloped ovaries that could not be assigned to “A” queens were coded “D” (with x the number of queens concerned). Finally, we considered if nestmate queens represented one or several haplotypic lineages.

| **ColID** | **NW** | **NQ** | **NDQ** | **NPDQ** | **NPM** | **NQM** | **NQWS** | **Code** |
| --- | --- | --- | --- | --- | --- | --- | --- | --- |
| **2** | 111 | 3 | 1 | 0 | 1 | 1 | 2 | A^c^(8)[C_2_] |
| **4** | 61 | 2 | 0 | 2 | 6 | 0 | 2 | A^b^(2)[C_2_]+A^p^(2)+A^p^(1)+A^f^(1)+A^f^(1)+A^p^(1) |
| **7** | 113 | 2 | 1 | 0 | 1 | 1 | 1 | A^c^(8)[C] |
| **8** | 113 | 8 | 4 | 0 | 5 | 1 | 6 | A^b^(3)[C]+A^b^(2)[B][C]+A^p^(1)[C]+A^c^(2)+A^p^[B_2_] |
| **15** | 37 | 3 | 2 | 0 | 3 | 1 | 1 | A^c^(6)[C]+A^p^(2)+A^c^(0) |
| **19** | 142 | 13 | 2 | 0 | 4 | 1 | 11 | A^c^(5)[C_4_]+A^b^(1)[C_7_]+A^p^(2)+A^c^(0) |
| **22** | 83 | 6 | 0 | 0 | 1 | 0 | 6 | A^b^(8)[C_6_] |
| **26** | 172 | 5 | 1 | 0 | 2 | 1 | 2 | A^c^(7)[C_2_]+A^p^(1)+D_2_ |
| **28** | 32 | 6 | 1 | 0 | 1 | 1 | 5 | A^c^(8)[C_5_] |
| **29** | 37 | 2 | 1 | 0 | 1 | 1 | 1 | A^c^(8)[C] |
| **30** | 36 | 5 | 3 | 0 | 4 | 2 | 1 | A^c^(5)+A^c^(2)[C]+A^p^(1)+A^c^(0)+D |
| **31** | 28 | 4 | 1 | 0 | 3 | 0 | 4 | A^b^(4)+A^b^(2)[B][C_3_]+A^p^(2) |
| **32** | 52 | 2 | 1 | 0 | 1 | 1 | 1 | A^c^(8)[C] |
| **33** | 365 | 11 | 2 | 0 | 3 | 2 | 9 | A^c^(2)[C_7_]+A^c^(4)[C_2_]+A^p^(2) |
| **34** | 41 | 8 | 5 | 0 | 5 | 0 | 5 | A^b^(4)[B_4_]+A^b^(2)[C]+A^f^(1)+A^p^(1)+A^c^(0)+D_2_ |
| **37** | 19 | 5 | 5 | 0 | 9 | 1 | 4 | A^b^(5)[B(2)+B_3_]+A^b^(3)+A^b^(3)+A^p^(2)+A^p^(1)  +A^p^(1)+A^p^(1)+A^p^(1)+A^c^(0)  *[all workers genotyped]* |
| **42** | 52 | 2 | 0 | 1 | 4 | 0 | 2 | A^b^(3)[C_2_]+A^p^(2)+A^p^(2)+A^p^(1) |
| **50** | 26 | 2 | 2 | 0 | 3 | 2 | 0 | A^c^(4)+A^b^(2)[B(2)] |
| **56** | 21 | 2 | 1 | 1 | 1 | 1 | 1 | A^c^(8)[C] |
| **57** | 17 | 2 | 1 | 0 | 6 | 1 | 1 | A^b^(8)[B]+A^b^(4)[C]+A^p^(2)+A^p^(1)+A^p^(1)+A^p^(1)  *[all workers genotyped]* |
| **58** | 22 | 4 | 1 | 0 | 4 | 1 | 0 | A^b^(3)[C]+A^c^(2)+A^p^(2)+A^p^(1)[C]+D |
| **59** | 25 | 5 | 1 | 0 | 2 | 1 | 4 | A^c^(6)[C_4_]+A^p^(2) |
| **60** | 57 | 5 | 2 | 0 | 4 | 1 | 3 | A^c^(6)[C_3_]+A^p^(1)+A^p^(1)+A^c^(0) |
| **61** | 50 | 4 | 0 | 1 | 2 | 0 | 3 | A^b^(4)[C_2_]+A^b^(4)[C*]+D  * different haplotypic lineage |
| **62** | 130 | 2 | 1 | 0 | 2 | 1 | 1 | A^c^(5)+A^b^(3)[C] |
| **63** | 277 | 15 | 11 | 0 |  |  |  | not possible to conclude |
| **70** | 42 | 3 | 3 | 0 |  |  |  | not possible to conclude |
| **71** | 110 | 5 | 3 | 0 | 3 | 3 | 2 | A^c^(4)[C]+A^c^(3)[C]+A^c^(1) |
| **72** | 84 | 5 | 0 | 1 | 2 | 0 | 1 | A^b^(5)[C]+A^b^(3)+D_4_ |
| **73** | 18 | 2 | 2 | 0 | 3 | 2 | 0 | A^c^(4)+A^b^(3)+A^c^(1) |
| **74** | 149 | 7 | 4 | 0 | 4 | 3 | 4 | A^c^(4)[B(1),B]+A^c^(2)[C_2_]+A^p^(1)+D |
| **38** | 2 | 2 | 0 | 0 | 2 | 0 | 2 | A^b^(1)[C_2_]+A^p^(1) |
| **39** | 2 | 2 | 0 | 0 | 2 | 0 | 0 | A^p^(1)+A^p^(1)+D_2_*  * different haplotypic lineages |

**Table S2.** primer sequences. f = forward, r = reverse.

**Primer Sequence Source**

LXAGa1 f 5′ − TTG CTC CAC TTG CTC CAC AAC − 3′ Bourke et al. (1997)

LXAGa1 r 5′ − CGA GTC GCC GTC AAA ACC TAT C − 3′ Bourke et al. (1997)

LXAGa2 f 5′ − TTA GAC GGA ATC AGA AAC CC − 3′ Bourke et al. (1997)

LXAGa2 r 5′ − AAC GGA AAG TAG CGA TTA CG − 3′ Bourke et al. (1997)

L-18 f 5′ − TGA ATT TGG ATG GCG GTA GAC − 3′ Foitzik et al. (1997)

L-18 r 5′ − ACC TAA TGC ACG CTT TAG AAT − 3′ Foitzik et al. (1997)

LXGT218 f 5′ − GTT CTT GCG CGG ATG CAT AC − 3′ Hamaguchi et al. (1993)

LXGT218 r 5′ − TGT ACT CGC GTG TCT ATC GG − 3′ Hamaguchi et al. (1993)

LXGT223 f 5′ − AAA CAT AAT ATT CGC GCA TAT CCA − 3′ Hamaguchi et al. (1993)

LXGT223 r 5′ − AAA AGA GGG CGA CGC GCA TC − 3′ Hamaguchi et al. (1993)

2MS34 f 5′ − GGC GTG CAC TCT TAG GGG ACA − 3′ Suefuji et al. (2011)

2MS34 r 5′ − GCT ACA CGA TAA CGA TAT CGC − 3′ Suefuji et al. (2011)

2MS46 f 5′ − GCT CAC TAC TAT GCT GCC AGC − 3′ Suefuji, unpublished

2MS46 r 5′ − CCT TCC TGC AAA CCA CGT GT − 3′ Suefuji, unpublished

Ant10878 f 5′ − CGG GTG YTA GTC GTC GCC AT − 3′ Butler et al. (2014)

Ant10878 r 5′ − GAT CAA TGC CGC AAC GCT AA − 3′ Butler et al. (2014)

C1-J-2183 5’-CAACATTTATTTTGATTTTTTGG-3´ Simon et al. (1994)

Cw.3031 5´-TTTGCWCTMATCTGCCMTATT-3´ Simon et al. (1994)

COI-516for 5´-ATTTTTYTCTATATTTATYGGA-3´ Simon et al. (1994)

C2-N-3661 5´-CCACAAATTTCTGAACATTGACCA-3´ Simon et al. (1994)

**Table S3.** Detection of stuttering (Stutt), large allele dropout (Drop), Oosterhout null allele frequencies (Null) (Microchecker 2.2.3), sample size (N), number of alleles (Na) and effective alleles (Ne), observed and expected heterozygosity (Ho/He), fixation index (F) (GenAlEx) and probability from Chi-squared test of Hardy-Weinberg equilibrium (HWE) (Genepop 4.7.5) for each of the 8 microsatellite markers.

|  | **Stutt** | **Drop** | **Null** | **N** | **Na** | **Ne** | **I** | **Ho** | **He** | **F** | **HWE** |
| --- | --- | --- | --- | --- | --- | --- | --- | --- | --- | --- | --- |
| **LXAGa1** | No | no | -0,049 | 33 | 5 | 3,320 | 1,310 | 0,758 | 0,699 | -0,084 | 0.691 |
| **LXAGa2** | No | no | -0,042 | 33 | 18 | 13,120 | 2,713 | 1,000 | 0,924 | -0,083 | 1.000 |
| **L-18** | No | no | 0,051 | 33 | 7 | 3,939 | 1,543 | 0,667 | 0,746 | 0,106 | 0.036 |
| **LXGT218** | No | no | 0,040 | 33 | 3 | 1,847 | 0,756 | 0,424 | 0,459 | 0,075 | 0.358 |
| **LXGT223** | No | no | 0,049 | 33 | 13 | 3,642 | 1,713 | 0,667 | 0,725 | 0,081 | 0.219 |
| **2MS34** | No | no | 0,042 | 32 | 6 | 2,312 | 1,115 | 0,531 | 0,567 | 0,064 | 0.241 |
| **2MS46** | No | no | -0,045 | 33 | 5 | 2,498 | 1,079 | 0,636 | 0,600 | -0,061 | 0.201 |
| **Ant10878** | No | no | 0,043 | 32 | 6 | 2,322 | 1,130 | 0,531 | 0,569 | 0,067 | 0.206 |

**Table S4.** Linkage disequilibrium between each pair of the 8 microsatellite markers (Genepop 4.7.5).

| Locus#1 | Locus#2 | P-Value | S.E. |
| --- | --- | --- | --- |
| ------- | ------- | -------- | -------- |
| LXAGa1 | LXAGa2 | 0.3767 | 0.059425 |
| LXAGa1 | L-18 | 0.138510 | 0.028368 |
| LXAGa2 | L-18 | 1.000000 | 0.000000 |
| LXAGa1 | LXGT218 | 0.626430 | 0.017105 |
| LXAGa2 | LXGT218 | 0.534560 | 0.032592 |
| L-18 | LXGT218 | 0.270230 | 0.012518 |
| LXAGa1 | LXGT223 | 0.898720 | 0.023029 |
| LXAGa2 | LXGT223 | 0.686220 | 0.079909 |
| L-18 | LXGT223 | 0.882000 | 0.041805 |
| LXGT218 | LXGT223 | 0.445610 | 0.029993 |
| LXAGa1 | 2MS34 | 0.868210 | 0.019033 |
| LXAGa2 | 2MS34 | 0.586080 | 0.064440 |
| L-18 | 2MS34 | 0.404120 | 0.029322 |
| LXGT218 | 2MS34 | 0.275110 | 0.012623 |
| LXGT223 | 2MS34 | 0.717030 | 0.037149 |
| LXAGa1 | 2MS46 | 0.000000 | 0.000000 |
| LXAGa2 | 2MS46 | 0.328680 | 0.040098 |
| L-18 | 2MS46 | 0.262800 | 0.022325 |
| LXGT218 | 2MS46 | 0.709270 | 0.017915 |
| LXGT223 | 2MS46 | 0.375170 | 0.031955 |
| 2MS34 | 2MS46 | 0.806690 | 0.025231 |
| LXAGa1 | Ant10878 | 0.488440 | 0.031903 |
| LXAGa2 | Ant10878 | 0.206070 | 0.042931 |
| L-18 | Ant10878 | 0.449510 | 0.050702 |
| LXGT218 | Ant10878 | 0.630620 | 0.017803 |
| LXGT223 | Ant10878 | 0.595550 | 0.052925 |
| 2MS34 | Ant10878 | 0.075060 | 0.018843 |
| 2MS46 | Ant10878 | 0.263100 | 0.022088 |

**Table S5.** Probability to detect all of the two to three queens with balanced / unbalanced worker offspring. This calculation is based on the hypothesis that the variability of the 8 microsatellites markers is sufficient to identify the parental line for each worker (i.e., at least 1 allele differs between 2 workers from 2 different queens). To calculate the probability for detecting all the queens from colonies sired by 2 queens contributing to the offspring unequally, the following formula was used for each number of genotyped workers n between 5 and 15: P(2Q detected) = 1 – f_1_^n^ – f_2_^n^ where n is the number of genotyped workers, and f is the frequency of offspring. To calculate the probability for detecting all the queens in colonies sired by 3 queens contributing to the offspring equally, the following formula was used: P(3Q detected) = c/(a+b+c), where a is the number of option of successive drawings of workers leading to the detection of only 1 of the 3 queens, with $a=3$; b is the number of option of successive drawings leading to the detection of only 2 of the 3 queens with $b=3\times[2^{1}{+2}^{2}{+2}^{3}+\ldots+2^{n-1}]$; and c is the number of option of successive drawings leading to the detection all the 3 queens, with ${c= \sum_{i=1}^{n-2} [3}^{i+1}\times3\times[2^{1}{+2}^{2}{+2}^{3}+\ldots+2^{n-i-1}]]$.

|  |  | **number of genotyped workers** | | | | | | | | | | |
| --- | --- | --- | --- | --- | --- | --- | --- | --- | --- | --- | --- | --- |
|  |  | **5** | **6** | **7** | **8** | **9** | **10** | **11** | **12** | **13** | **14** | **15** |
| number of queens | 1 | *1* | *1* | *1* | ***1*** | *1* | *1* | *1* | *1* | *1* | *1* | *1* |
|  | 2 (50/50 offspring) | *0,94* | *0,97* | *0,98* | ***0,99*** | *1* | *1* | *1* | *1* | *1* | *1* | *1* |
|  | 2 (75/25 offspring) | *0,76* | *0,82* | *0,86* | ***0,89*** | *0,92* | *0,94* | *0,96* | *0,97* | *0,98* | *0,98* | *0,99* |
|  | 2 (90/10 offspring) | *0,45* | *0,47* | *0,52* | ***0,57*** | *0,61* | *0,65* | *0,69* | *0,72* | *0,75* | *0,77* | *0,79* |
|  | 3 | *0,62* | *0,74* | *0,83* | ***0,88*** | *0,92* | *0,95* | *0,97* | *0,98* | *0,99* | *0,99* | *1* |


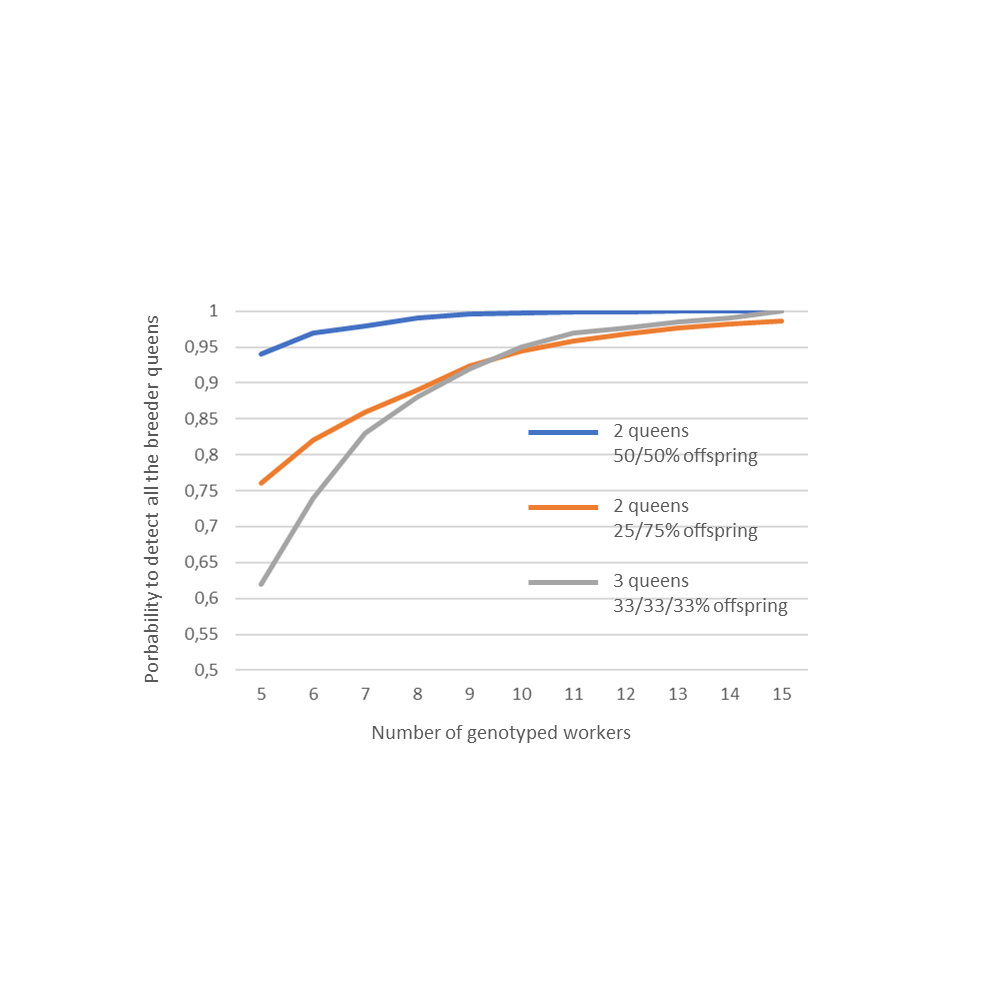


**Table S6.** Effects of nest type on: M1: the number of queens with developed ovaries, M2: the number of inseminated queens, M3: the number of workers in the colony, and M4: the relatedness between workers based on the Queller and Goodnight estimator.

| **Model 1: Nb. of developed queens** | **estimate** | **z.ratio** | **p-value** |  |  |
| --- | --- | --- | --- | --- | --- |
| Nest type A vs Nest type B | -1.810 | -2.403 | 0.049 |  |  |
| Nest type A vs Nest type C | -0.754 | -1.638 | 0.203 |  |  |
| Nest type B vs Nest type C | 1.056 | 1.445 | 0.203 |  |  |
| **Model 2: Nb. of Workers** | **estimate** | **t.ratio** | **p-value** |  |  |
| Nest type A vs Nest type B | -53.19 | -1.360 | 0.370 |  |  |
| Nest type A vs Nest type C | 5.59 | 0.178 | 0.860 |  |  |
| Nest type B vs Nest type C | 58.78 | 1.773 | 0.261 |  |  |
| **Model 3: Worker’s relatedness** | **estimate** | **t.ratio** | **p-value** |  |  |
| Nest type A vs Nest type B | 0.324 | 3.647 | 0.002 |  |  |
| Nest type A vs Nest type C | 0.405 | 5.707 | <0.001 |  |  |
| Nest type B vs Nest type C | 0.082 | 1.089 | 0.286 |  |  |

**Table S7.** Pairwise Wilcoxon rank sum tests between all pairs of queen types (n=15 tests). Above diagonal: W values. Below diagonal: p-values (grey: before Holm correction, black: after Holm correction).

| p\W | 1-1 | 1-2 | 1-3 | 2-2 | 2-3 | 3-3 |
| --- | --- | --- | --- | --- | --- | --- |
| 1-1 | - | 177 | 276 | 90 | 537 | 495 |
| 1-2 | 0.356 1.000 | - | 1632 | 658 | 3410 | 2974 |
| 1-3 | 0.986 1.000 | 0.410 1.000 | - | 732 | 3846 | 3558 |
| 2-2 | 0.021 0.187 | 0.008 0.080 | 0.001 **0.009** | - | 3500 | 3565.5 |
| 2-3 | 0.824 1.000 | 0.246 1.000 | 0.924 1.000 | <0.001 **0.002** | - | 6769.5 |
| 3-3 | 0.093 0.746 | 0.005 0.055 | 0.001 **0.009** | 0.431 1.000 | <0.001 **<0.001** | - |


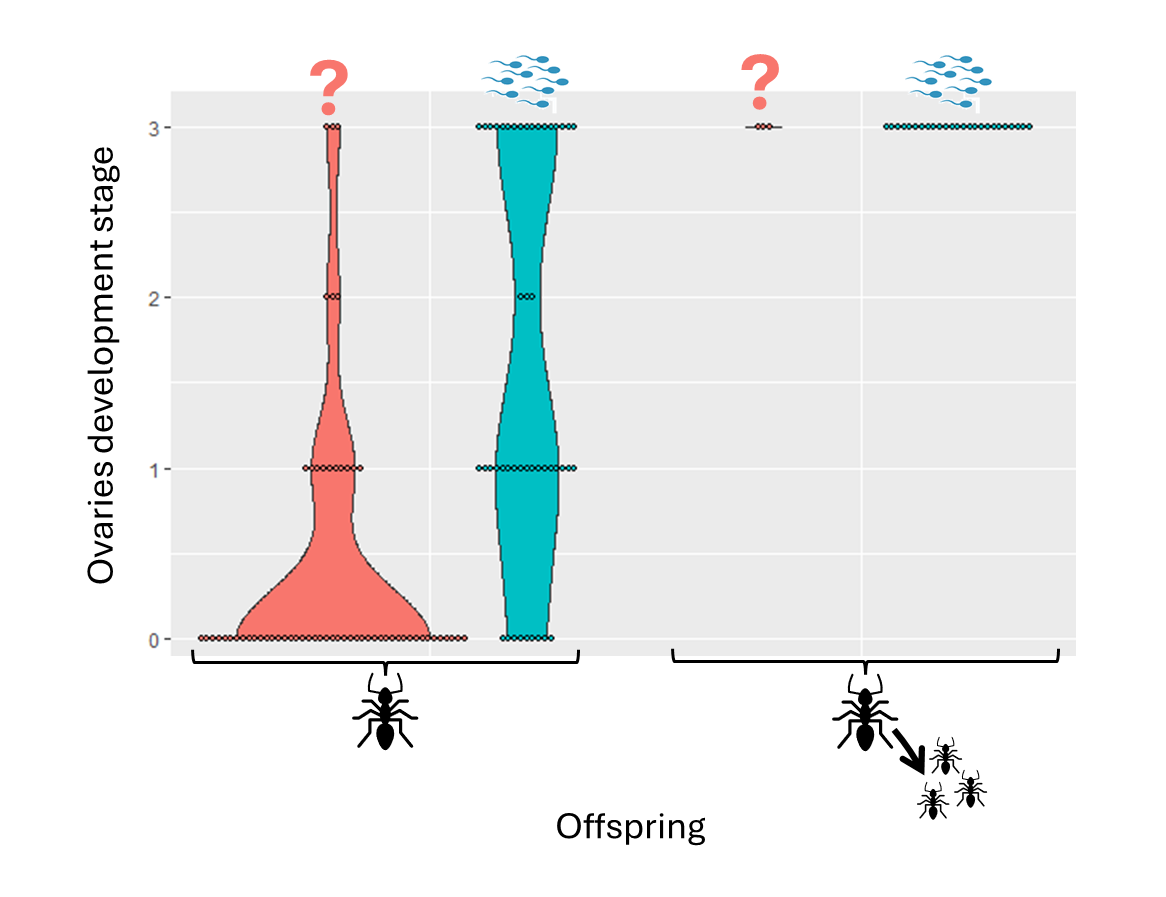


**Figure S1.** Content of the spermatheca (red: empty or uncertain, blue: filled) depending on offspring detection in queens with ovaries presenting different development stages. 0 (undeveloped), ovarioles not elongated, no developing eggs; 1 (hardly developed), ovarioles not elongated, 1-2 developing eggs; 2 (developing ovaries), ovarioles started to elongate, 2 or more developing eggs; 3 (fully developed), ovarioles fully elongated, yellow bodies present (modified from Heinze et al. 1992). Left: no offspring detected, right: offspring detected.


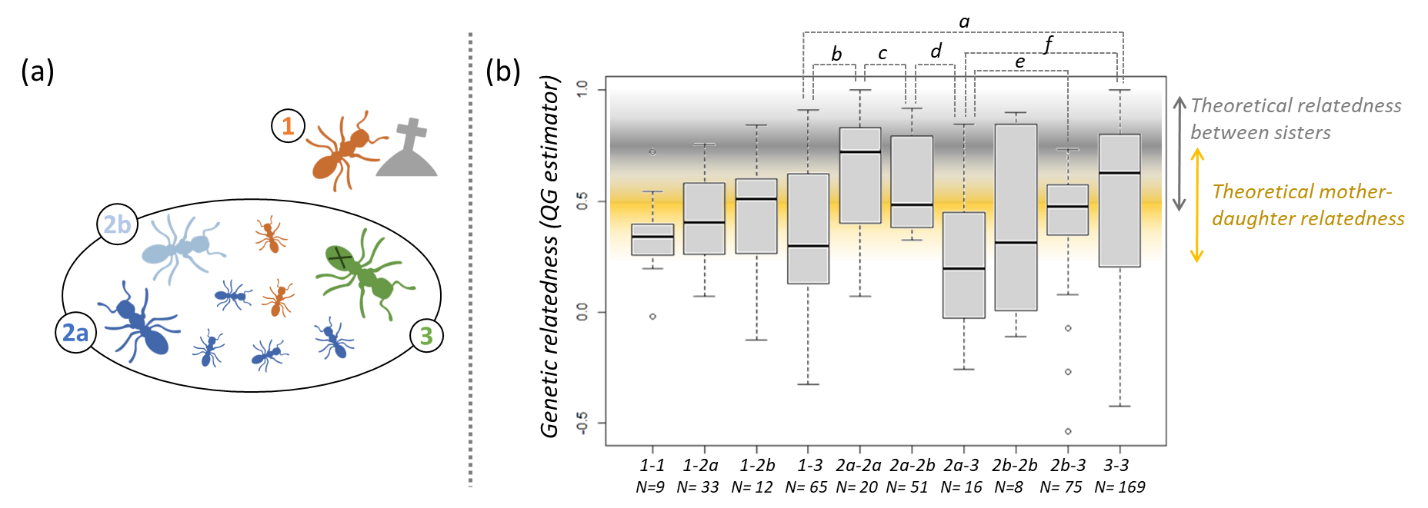


**Figure S2.** (a) Different types of queens identified in the study: 1: former mothers , not present in the nest; 2a: queens with developed ovaries without offspring; 2b: queens with developed ovaries with offspring; 3: queens without developed ovaries. (b) Genetic relatedness calculated between pairs of queens from the same nest. Significant differences between type of comparisons are indicated by letters. a: estimate = -0.167 ; t.ratio = -3.703; p.value = 0.010; b: estimate = -0.280; t.ratio = -3.620; p.value = 0.013; c: estimate = 0.420 ; t.ratio = 5.318; p.value < 0.001; d: estimate = 0.361 ; t.ratio = 3.755; p.value = 0.008; e: estimate = -0.222 ; t.ratio = -4.084; p.value = 0.002; f: estimate = -0.306 ; t.ratio = -6.422; p.value = < 0.001.


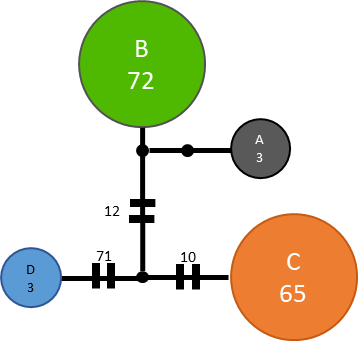


**Figure S3.** Maximum parsimony spanning network reconstructed with TCS based on 143 mtDNA Cox1-Cox2 contig sequences (1303bp). Each line represents a substitution, dots represent single missing haplotype, double bars with values indicates multiple missing haplotypes; size of the haplotypes proportional to the number of individuals with number of specimens indicated if superior at one.
